# Collateral Crises of Gun Preparation and the COVID-19 Pandemic: Infodemiology Study

**DOI:** 10.2196/19369

**Published:** 2020-05-28

**Authors:** Theodore L Caputi, John W Ayers, Mark Dredze, Nicholas Suplina, Sarah Burd-Sharps

**Affiliations:** 1 Department of Health Sciences University of York York United Kingdom; 2 Division of Infectious Diseases and Global Health Department of Medicine University of California San Diego San Diego, CA United States; 3 Department of Computer Science Johns Hopkins University Baltimore, MD United States; 4 Everytown for Gun Safety New York, NY United States

**Keywords:** COVID-19, gun, firearm, surveillance, injury

## Abstract

**Background:**

In the past, national emergencies in the United States have resulted in increased gun preparation (ie, purchasing new guns or removing guns from storage); in turn, these gun actions have effected increases in firearm injuries and deaths.

**Objective:**

The aim of this paper was to assess the extent to which interest in gun preparation has increased amid the coronavirus disease (COVID-19) pandemic using data from Google searches related to purchasing and cleaning guns.

**Methods:**

We fit an Autoregressive Integrated Moving Average (ARIMA) model over Google search data from January 2004 up to the week that US President Donald Trump declared COVID-19 a national emergency. We used this model to forecast Google search volumes, creating a counterfactual of the number of gun preparation searches we would expect if the COVID-19 pandemic had not occurred, and reported observed deviations from this counterfactual.

**Results:**

Google searches related to preparing guns have surged to unprecedented levels, approximately 40% higher than previously reported spikes following the Sandy Hook, CT and Parkland, FL shootings and 158% (95% CI 73-270) greater than would be expected if the COVID-19 pandemic had not occurred. In absolute terms, approximately 2.1 million searches related to gun preparation were performed over just 34 days. States severely affected by COVID-19 appear to have some of the greatest increases in the number of searches.

**Conclusions:**

Our results corroborate media reports that gun purchases are increasing amid the COVID-19 pandemic and provide more precise geographic and temporal trends. Policy makers should invest in disseminating evidence-based educational tools about gun risks and safety procedures to avert a collateral public health crisis.

## Introduction

Collateral threats to population health from the coronavirus disease (COVID-19) pandemic should not be ignored. COVID-19 has effected political and economic uncertainty worldwide, and the World Health Organization has called for public health practitioners to monitor health hazards that may emerge from individuals’ attempts to cope with these exceptional circumstances [1].

Firearm injuries may be one such hazard. Citing examples of past emergencies [2], it has been suggested that individuals will respond to the uncertainty of the COVID-19 pandemic by preparing guns, including buying new guns or removing guns from lockers or other storage units. The link between gun access and unintentional firearm injury/death is well established in the literature. For example, Bangalore and Messerli [3] used survey and administrative data from 27 developed countries and found a significant correlation between the number of guns per capita in a country and the rate of firearm-related deaths (*P*<.001). Within the United States, Miller et al [4] found a strong and robust correlation between gun availability and unintentional firearm deaths at the state level. For example, they estimated that the risk of unintentional firearm death in US states with the highest level of gun availability was approximately nine times that in states with the lowest level of gun availability.

Unfortunately, traditional surveillance of gun preparation is limited [5]. Since 1996, the US Congress has dramatically restricted the ability of the National Institutes of Health and the Centers for Disease Control and Prevention to conduct gun violence research. This constraint was recently eased; however, funding for this research still only totals $25 million [6]. The main source of public data related to legal gun sales is monthly reports from the Federal Bureau of Investigation (FBI) National Instant Criminal Background Check System (NICS); however, these reports only represent background checks, not sales, and they are only available on a monthly basis at nationally aggregated and state levels. Many of these background checks are conducted for purposes other than new gun sales (ie, permit renewals). Further, these data may be unhelpful to local stakeholders who are hoping to respond to weekly or even daily changes in the sentiment surrounding guns. Finally, this system only accounts for people buying guns from sellers who require a background check; it does not include online firearms sales from private sellers, sales at gun shows, or illegal purchases. The uncertainty and rapidly changing circumstances of a pandemic such as COVID-19 only amplify the limitations of traditional data, as policy makers and other stakeholders have limited time to design and implement interventions before permanent damage to population health is incurred.

Consequently, we turn to infodemiology [7-9], a field defined as “the science of distribution and determinants of information in an electronic medium, specifically the Internet, or in a population, with the ultimate aim to inform public health and public policy” [10]. Infodemiology is particularly useful in situations where relevant traditional data is not readily available, such as when researchers wish to provide a timely response to an epidemic [11-13] or to predict emerging population health concerns [14].

One prominent infodemiology tool that has been used frequently in public health as well as in other gun control research is Google Trends [15], a web application and application programming interface (API) that allows users to provide a set of keywords and a timeframe of interest and retrieve the proportion of all Google searches containing those keywords over that timeframe. Google Trends has become an important data source for studies in public health surveillance generally [16] and for gun violence research in particular. For example, in past studies, Google Trends was used to assess the effect of mass shooting incidents on public interest in gun control [17], to approximate gun ownership [18], and to predict gun purchases [19] and firearm injuries [20]. In this study, we used Google Trends to assess gun preparation amid the COVID-19 pandemic.

## Methods

### Data

We extracted weekly data on all US Google searches (including state-level data) related to gun preparation, that is, searches that contain the terms “buy gun(s)” or “clean gun(s)” (eg, “how to clean gun” or “where to buy guns”), executed between January 4, 2004 and April 11, 2020 from the Google Trends for Health API. That is, we programmatically queried the Google Trends for Health API for the United States and for each individual US state for weekly data regarding searches matching any combination of those two lists between January 1, 2004 and April 12, 2020. The code used to pull this data from the Google Trends for Health API is available from the authors upon request. The API reports these data as “query fractions,” or the fraction of all Google searches that include the focal terms, thereby accounting for differences in overall Google usage over time and across locations.

### Statistical Analysis

We first described trends in US gun preparation searches. We extrapolated query fractions to raw count estimates using publicly available data from Comscore [21]. Specifically, we assumed that the number of searches remained at the level of the most recently available data (February 2020), that searches were conducted uniformly throughout the month, and that desktop searches represented 35% of all searches. Using these assumptions, we calculated estimates for the number of Google searches per day, which allowed us to extrapolate the number of searches related to gun preparation from the Google Trends query fraction. Although this method only provides a rough estimate, it is a common approach in the Google Trends health literature [22].

Next, we fit an Autoregressive Integrated Moving Average (ARIMA) model using the Hyndman-Khandakar algorithm [23] over all US query fraction values up to March 7, 2020. We chose this cutoff because US President Donald Trump declared COVID-19 a national emergency on March 13, 2020, which is included in the data for the following week. We forecasted query fraction values for the United States from March 8, 2020 to April 11, 2020 and reported the difference between the actual and forecasted values. Finally, we calculated the percentage change ((after – before)/before × 100%) in the mean query fractions before and after the onset of the pandemic (using January 1, 2020 to March 7, 2020 as the preperiod) for each state with bootstrapped confidence intervals. We used a univariate linear regression and data from USAFacts.org [24] to calculate the correlation between this percentage change and the number of COVID-19 deaths per capita in the postperiod by state.

Analyses were conducted using R version 3.6.3 (R Foundation) with α=.05.

## Results

**Figure 1:**
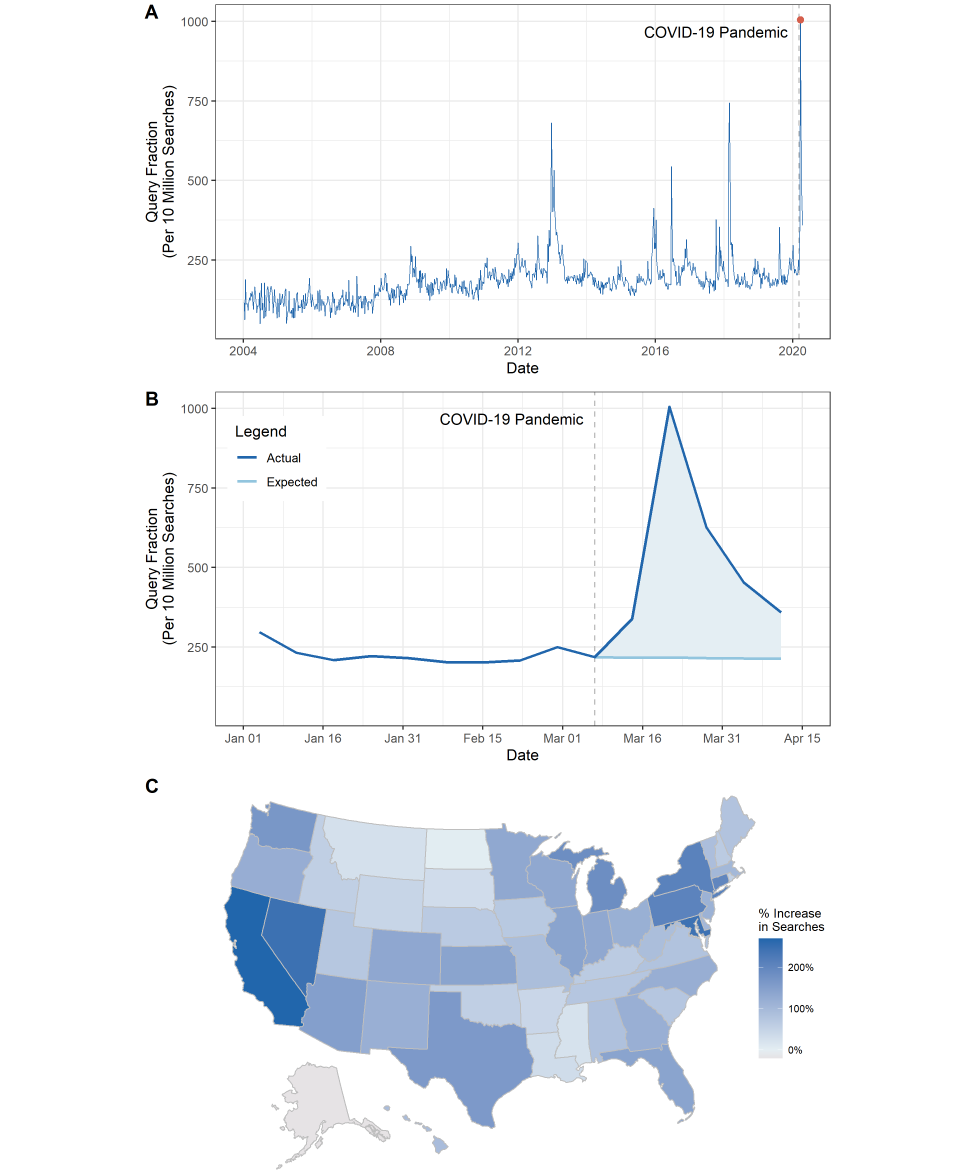
Google searches for gun preparation before and during the COVID-19 pandemic. A) The fraction of Google search queries that relate to gun preparation between January 1, 2004 and April 11, 2020. B) The fraction of Google search queries that relate to gun preparation between January 1, 2020 and April 11, 2020. C) The percentage change in the query fractions for a preperiod between January 1, 2020 and March 7, 2020 and a postperiod between March 8, 2020 and April 11, 2020.

By March 21, 2020, approximately 1000 of every 10 million Google searches were related to gun preparation. For reference, this query fraction is 35% and 48% greater than the spikes occurring after the mass shootings in Sandy Hook, Connecticut in 2012 and in Parkland, Florida in 2018, respectively (Figure 1A). The fraction of Google searches related to gun preparation significantly (*P*<.05) exceeded the ARIMA-forecasted values for each week since President Trump declared a national emergency. Approximately 2.1 million gun preparation searches were executed between March 8 and April 11, 2020, which is 158% greater (95% CI 73-270) than would be expected if the COVID-19 pandemic had not occurred.

Figure 1A shows the fraction of Google search queries that relate to gun preparation between January 1, 2004, the first date for which data is available, and April 11, 2020. The blue line is the actual fraction of Google searches. The dotted vertical line is placed at March 7, 2020, denoting the breakpoint between the preperiod and the postperiod, which was chosen based on the week in which President Trump declared COVID-19 a national emergency. Figure 1B shows the fraction of Google search queries that relate to gun preparation between January 1, 2020 and April 11, 2020. The dark blue line is the actual fraction of Google searches. The light blue line represents the expected fraction of Google searches based upon the ARIMA model fitted over data from January 1, 2004 to March 7, 2020. The dotted vertical line is placed at March 7, 2020. The shaded area represents excess searches (ie, searches in excess of the number forecasted by the ARIMA model). Figure 1C shows the percentage change in the query fractions for the preperiod between January 1, 2020 and March 7, 2020 and the postperiod between March 8, 2020 and April 11, 2020.

**Figure 2:**
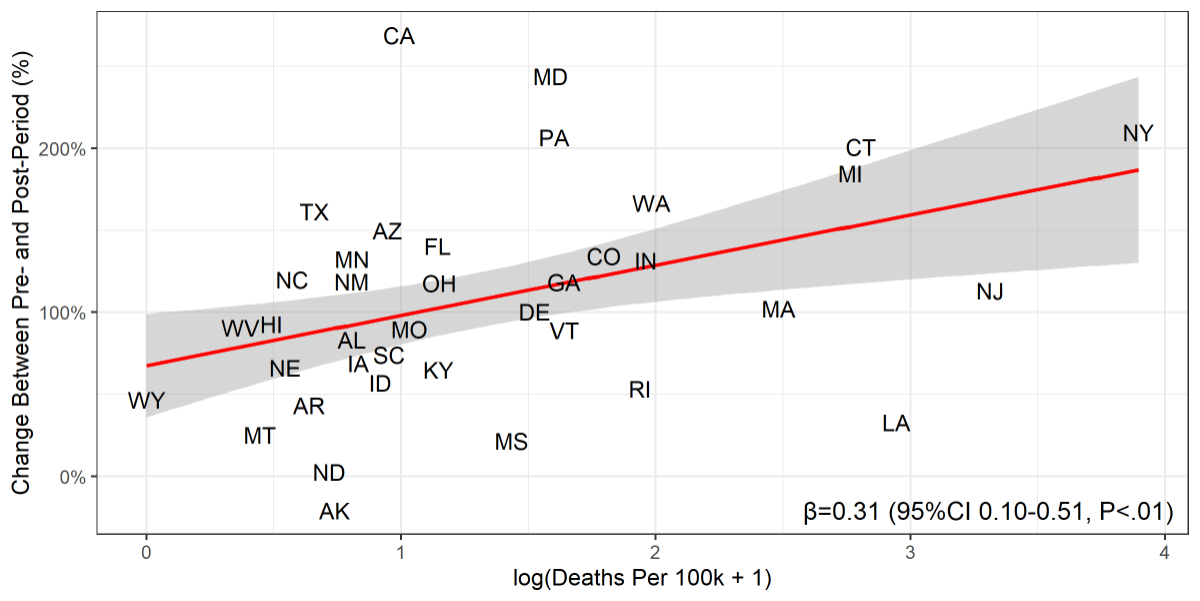
State-level correlation between the COVID-19 death rate and the percentage increase in gun preparation searches.

Forty-nine states (all but Alaska) and the District of Columbia experienced increases in gun preparation searches. The states most affected by the pandemic in the early period examined in this study appear to have particularly high percentage changes in searches, including California (269%, 95% CI 120-477), New York (210%, 95% CI 81-380), Connecticut (201%, 95% CI 90-356), and Washington (167%, 95% CI 85-262). In an ecological, state-level univariate regression, we found that a 1 percent increase in the COVID-19 death rate (ie, COVID-19 deaths per 100,000) was correlated at the state level with an approximately 0.31 higher percent change (95% CI 0.10-0.51) in gun preparation searches. Figure 2 shows the correlation between the natural logarithm of the number of COVID-19 deaths per 100,000 people occurring during the postperiod (plus 1 to correct for infinite values) and the percentage change in gun preparation searches between the preperiod and the postperiod. The preperiod is defined as the dates between January 1, 2020 and March 7, 2020 and the postperiod is defined as dates between March 8, 2020 and April 11, 2020. The labels represent individual states, the red line represents a univariate linear regression model, the gray area represents the confidence interval for that univariate regression model, and the parameter estimates in the bottom right corner refer to the results of that model.

## Discussion

### Principal Findings

Public interest in gun preparation has reached unprecedented levels amid the COVID-19 pandemic, approximately 40% greater than the spikes occurring after the Sandy Hook and Parkland, Florida shootings. Increases in interest appear to be concentrated in the areas most affected by COVID-19.

This study demonstrates the value that Google search data presents to policy makers, regulators, advocates, and other stakeholders for surveillance of gun preparation. Public health professionals must be able to nimbly respond to the changing and frequently diverse health needs of the public. Google search data is timelier and has better temporal and geographic precision than the administrative data available for gun violence in the United States, and public health professionals should leverage these advantages so they can respond to the public at the exact times and in the exact states that are necessary. For example, our study shows that Google searches related to preparing guns are still elevated relative to expectations.

### Limitations

**Figure 3:**
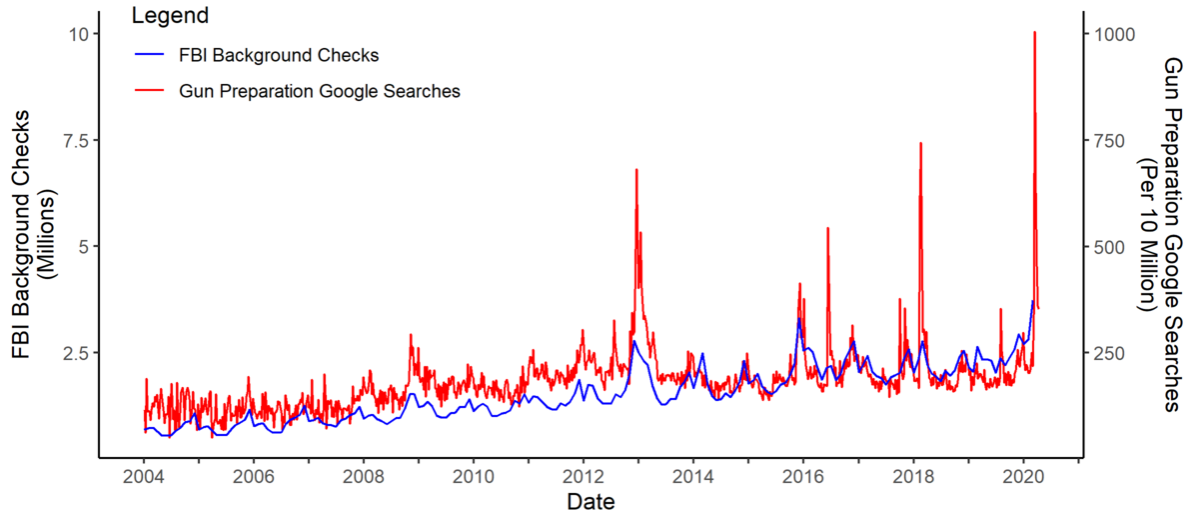
Correlation between gun preparation Google searches and FBI background checks. The weekly gun preparation Google searches (red line) are overlaid with the monthly numbers of background checks provided by the FBI National Instant Criminal Background Check System. FBI: Federal Bureau of Investigation.

This study has limitations. We only observed the volume of Google searches related to gun preparation, not the motivation for each search. All studies using aggregate Google searches are limited in that it is impossible to observe the etiology of a search. However, a previous study found that search volumes using these exact search terms significantly predicted both gun purchases and firearm injuries/deaths [20], and we observed similar trends between these searches and FBI NICS estimates in past periods (Figure 3); this increases our confidence that these searches will predict similar outcomes. Additionally, studies across several public health domains have demonstrated that Google searches can predict traditional surveillance metrics [25-27]. Analytically, our state-level correlation is an ecological analysis and does not imply an individual-level correlation between COVID-19 deaths and gun preparation [28].

### Conclusions

Given the well-established association between access to guns and firearm injuries, this surge in interest may compound the health risks of the COVID-19 pandemic.

Gun safety organizations, such as Everytown for Gun Safety, have created evidence-based materials and programs to educate the public on the risks of owning a firearm and the necessary safety precautions responsible gun owners should take to reduce the risk to themselves and their families [29]. Our results represent a call to action for policy makers, advocates, and public health officials to invest in educating the public and broadly disseminating these materials.

## Abbreviations

API: application programming interface
ARIMA: Autoregressive Integrated Moving Average
COVID-19: coronavirus disease
FBI: Federal Bureau of Investigation
NICS: National Instant Criminal Background Check System
